# Photoactivatable Heptamethine-Based Carbonic Anhydrase Inhibitors Leading to New Anti-Antibacterial Agents

**DOI:** 10.3390/ijms24119610

**Published:** 2023-06-01

**Authors:** Simone Carradori, Andrea Angeli, Patrick S. Sfragano, Xheila Yzeiri, Massimo Calamante, Damiano Tanini, Antonella Capperucci, Hannah Kunstek, Mihayl Varbanov, Clemente Capasso, Claudiu T. Supuran

**Affiliations:** 1 Department of Pharmacy, “G. d’Annunzio” University of Chieti-Pescara, 66100 Chieti, Italy; simone.carradori@unich.it; 2 Neurofarba Department, Sezione di Scienze Farmaceutiche, University of Florence, Via Ugo Schiff 6, Sesto Fiorentino, 50019 Florence, Italy; 3 Department of Chemistry “Ugo Schiff”, University of Florence, Via Della Lastruccia 3-13, 50019 Sesto Fiorentino, Italy; 4 CNR-Institute of Chemistry of Organometallic Compounds (CNR-ICCOM), Via Madonna del Piano 10, 50019 Sesto Fiorentino, Italy; 5 Department of Biotechnology, Chemistry and Pharmacy, University of Siena, 53100 Siena, Italy; 6 Institute of Molecular Biotechnology, Graz University of Technology, 8010 Graz, Austria; 7 L2CM, Centre National de la Recherche Scientifique (CNRS), Université de Lorraine, 54000 Nancy, France; 8 Laboratoire de Virologie, Centres Hospitaliers Régionaux Universitaires (CHRU) de Nancy Brabois, 54500 Vandœuvre-lès-Nancy, France; 9 Department of Biology, Agriculture and Food Sciences, National Research Council (CNR), Institute of Biosciences and Bioresources, 80131 Naples, Italy

**Keywords:** carbonic anhydrase, *S. epidermidis*, antimicrobial, photodynamic therapy, indocyanine

## Abstract

With the aim to propose innovative antimicrobial agents able to not only selectively inhibit bacterial carbonic anhydrases (CAs) but also to be photoactivated by specific wavelengths, new heptamethine-based compounds decorated with a sulfonamide moiety were synthesized by means of different spacers. The compounds displayed potent CA inhibition and a slight preference for bacterial isoforms. Furthermore, minimal inhibitory and bactericidal concentrations and the cytotoxicity of the compounds were assessed, thus highlighting a promising effect under irradiation against *S. epidermidis*. The hemolysis activity test showed that these derivatives were not cytotoxic to human red blood cells, further corroborating their favorable selectivity index. This approach led to the discovery of a valuable scaffold for further investigations.

## 1. Introduction

The development of new antibacterial treatments became a worldwide priority in the last decade due to increasingly frequent drug resistance phenomena among all the pathogen microorganism strains. Unfortunately, the lack of new classes of antibiotics hinders finding efficacious treatments due to multidrug resistance (MDR) to the principal classes of antibiotics on the market to date [1]. Indeed, the World Health Organization (WHO) in a 2021 report described the antibacterial clinical and preclinical pipeline as stagnant and far from meeting global needs, with only two antibiotics approved in the last five years with an innovative mechanism of action to overcome antimicrobial resistance [2]. The development of new drugs to combat resistant pathogens has mainly focused on the modification of existing compounds, leading to a decline in the drug discovery process. However, non-traditional approaches offer new opportunities to tackle infections from resistant bacteria as they can be used complementarily and synergistically or as alternatives to established therapies. Within the emerging antimicrobial therapeutic arsenal, light-based approaches have shown particular promise [3,4]. This process takes advantage of light and light-sensitive agents (photosensitizers) in an oxygen-rich environment, where there is a transfer of energy from photons to the photosensitizer. This can lead to the generation of harmful reactive oxygen species from the neighboring molecules that damages bacterial biomacromolecules (proteins, DNA, RNA, and lipids). The most important determinant in this approach is the selection of the photosensitizer such as synthetic and natural dyes (methylene blue, toluidine blue, tetrapyrrolic structures, curcumin, hypericin). Among them, heptamethines, belonging to the cyanine dye family, have been developed in biomedical imaging and theranostic production.

The versatility of photodynamic therapy (PDT) can be highlighted against bacteria involved in hard-to-treat infections, especially those forming the ESKAPE group and, in addition, the opportunity for microorganisms to develop resistance by selecting mutant strains is supposed to be very limited [5,6,7]. The indocyanine scaffold is a well-known near-infrared fluorescent dye photosensitizer that has been used in optical imaging and photothermal therapy; however, this class of derivatives has not shown a specific biological target, leading to their limited use [8].

In this context, a novel and promising anti-infective target is the carbonic anhydrase (CA, E.C. 4.2.1.1) enzyme from different microorganism species. The CA superfamily contains eight genetically distinct families (or classes), named α-, β-, γ-, δ-, ζ-, η-, θ-, and ι-CAs [9]. However, all CA classes catalyze the same reaction of CO_2_ hydration into a bicarbonate anion and proton, which is essential for the growth and pathogenicity of the microbes carried out by the CAs belonging to the α-, β-, γ- and ι-CA classes [10].

Recent successes have been observed in identifying carbonic anhydrase inhibitors (CAIs) with antibacterial activity [11,12], and their employment could open new scenarios in this field. For this reason, the conjugation of a CAI with an indocyanine moiety could be useful to bypass the emergence of new drug resistance phenomena. Considering what was mentioned above, the purpose of the current study was to evaluate the antimicrobial effect of carbonic anhydrase inhibitors in combination with photodynamic therapy against different strains of bacteria.

## 2. Results and Discussion

### 2.1. Compounds Design and Synthesis

**Figure 1:**
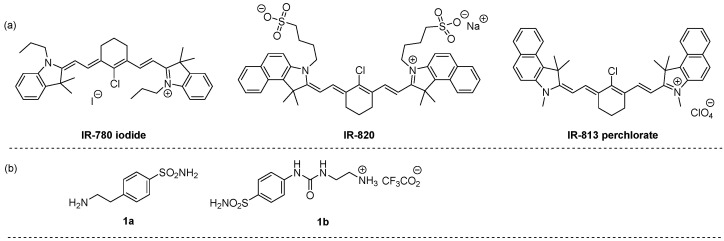
Structure of near-infrared lipophilic cation heptamethine fluorescent dyes (**a**) and functionalized amines (**b**) used in this work.

Keeping in mind compounds with the general features of systems 2, their synthesis was approached by exploiting the reactivity of benzenesulfonamide derivatives bearing a nucleophilic aminic function with suitable indocyanine-related electrophilic partners. The use of various nucleophiles bearing the benzenesulfonamide moiety has been indeed harnessed to access a broad range of structurally diverse carbonic anhydrase inhibitors [13,14,15]. In this regard, our attention was attracted by the possibility to functionalize near-infrared lipophilic cation heptamethine fluorescent dyes, namely IR-780 iodide, IR-820, and IR-813 perchlorate, bearing a C-Cl bond (Figure 1a). The nucleophilic replacement of chlorine with amines bearing the benzenesulfonamide moiety (Figure 1b) would enable the synthesis of novel indocyanine-derived near-infrared fluorescent dye photosensitizer hybrids with carbonic anhydrase inhibitor activity.

**Scheme 1:**
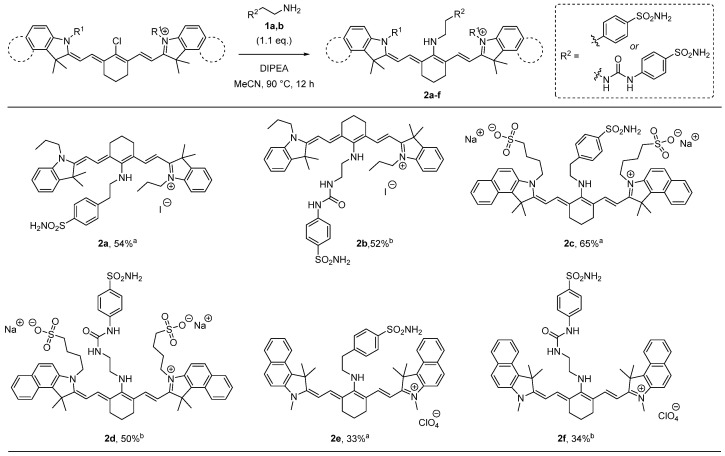
Synthesis of compounds **2a**–**f**. ^a^ 1.5 eq. of DIPEA was used. ^b^ 3.0 eq. of DIPEA was used. Yields refer to purified products.

Related transformations employing simple amines are well documented in the literature. The replacement of chlorine with N-centered nucleophiles is generally promoted by tertiary amines such as triethylamine (Et_3_N) and *N*,*N*-diisopropylethylamine (EtN*^i^*Pr_2_; DIPEA). Acetonitrile is generally the solvent of choice for these reactions [16,17]. Based on these considerations, the synthesis of the target compounds was investigated upon treatment of IR-780 iodide with **1a** using both Et_3_N and DIPEA in acetonitrile under different reaction conditions of temperature (ambient temperature and 90 °C) and time (6 h, 12 h, and 24 h). In our hands, the use of DIPEA in acetonitrile under refluxing conditions for 12 h led to the formation of derivative **2a** in higher yield. Thus, the preparation of compounds **2a**–**f** was pursued by reacting the electrophilic near-infrared lipophilic cation heptamethine fluorescent dyes reported in Figure 1 with functionalized amines **1a**,**b** under these optimized conditions as reported in Scheme 1. Remarkably, these conditions also worked well in the case of the ureido-substituted ammonium trifluoroacetate derivative **1b**, which required the use of a larger excess of base. The protocol could be efficiently applied to the three studied dyes, leading to the formation of the corresponding benzenesulfonamide-containing hybrids having iodide **2a**,**b**, sulfonate **2c**,**d**, and perchlorate **2e**,**f** as counterions (Scheme 1).

### 2.2. UV–Vis Absorption and Fluorescent Emission Spectra

**Figure 2:**
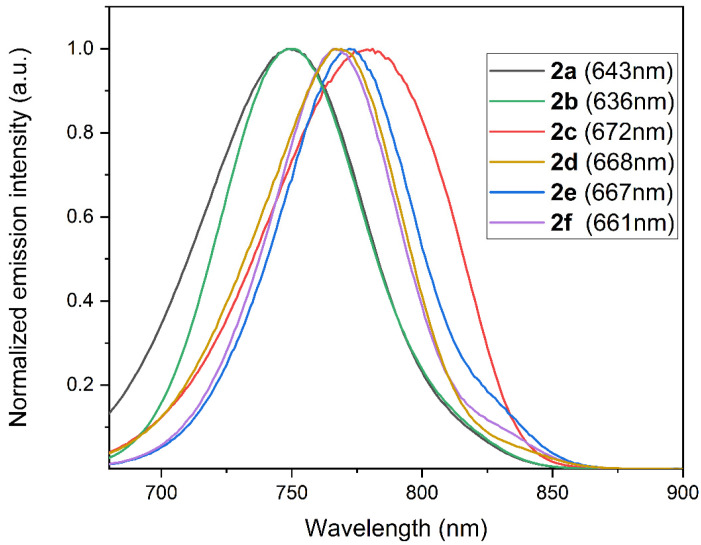
Normalized fluorescence emission spectra of compounds **2a**–**f** (excitation wavelengths in brackets) in a methanol solution (concentrations: **2a**, 2.4 × 10^−6^ M; **2b**, 3.1 × 10^−6^ M; **2c**, 2.3 × 10^−6^ M; **2d**, 6.2 × 10^−6^ M; **2e**, 4.0 × 10^−6^ M; **2f**, 5.3 × 10^−6^ M).

The compounds **2a**–**f** were characterized by UV–Vis spectroscopy and fluorescence spectroscopy. The measurements were made in a methanol solution. All compounds showed UV–Vis and fluorescence spectra characteristic of cyanins [18]. In particular, compounds **2a**–**f** were characterized by an absorption maximum between 640 and 670 nm (Figures S1–S6, ESI), while compounds **2d**–**f** showed a further absorption maximum between 810 and 820 nm. The fluorescence spectrum obtained by excitation of the absorption bands at 640–670 nm showed intense emission peaks with maximums between 743 and 752 nm (Figure 2).

### 2.3. Carbonic Anhydrase Inhibition

Compounds **2a**–**f** were first assayed in vitro against the physiologically off-target human CA isoforms such as hCAs I, II, and the bacterial isoforms from *Mammaliicoccus sciuri* (MsCA), *Escherichia coli* (*E. coli* β and γ), *Pseudomonas aeruginosa* (PsCA1 and PsCA3), and *Streptococcus mutans* (SmuCA) by applying the stopped-flow technique, and are compared in Table 1 with the standard sulfonamide inhibitor acetazolamide (**AAZ**). The CA isoforms belong to different classes of CA enzymes characterized by different kinetic and structural features to assess isoform selectivity.

The enzymatic assay displayed weak inhibition against the off-target hCA I with *K*_I_ values in the high nanomolar range (*K*_I_ 410–957.1 nM), and for **2f**, a value in the micromolar range was observed (*K*_I_ 3899 nM). In a similar manner, all tested compounds had *K*_I_ values in the medium/high nanomolar range for the second isoform off-target hCA II, whereas **AAZ** was a potent inhibitor of this isoform. However, these compounds were more active towards this isoform than hCA I. Notably, all the bacterial isoforms showed greater inhibition values compared to those of the two human off-target CA isoforms. In particular, compounds **2b** and **2f** were near 10-fold more selective for all assayed bacterial isoforms. On the other hand, among the beta and gamma classes of *E. coli* it was possible to observe, in general, a slight difference in the inhibition values of the synthesized compounds, which showed two-fold better *K*_I_s towards the β-class than the γ one. Generally, all compounds displayed better inhibition against the bacterial isoforms than the off-target hCA I and II, and among the bacterial Cas, these compounds did not show particular selectivity towards one of them. Finally, the effect of the counterion in this scaffold was not so relevant for inhibitory activity.

### 2.4. Antibacterial Assays

In light of the results discussed above and the improved affinity towards the β-class of bacterial Cas, not expressed in humans, their minimal inhibitory concentration (MIC) was evaluated against three Gram-positive bacteria such as *E. faecalis*, *S. aureus*, and *S. epidermidis*, and the data are reported in Table 2.

Among the synthesized compounds, **2c** and **2d** did not show antibacterial effects against all the strains considered over 256 μg/mL (they were also characterized by the same counterion). On the other hand, **2a**, **2b**, **2e**, and **2f** showed moderate activity against the *E. faecalis* and *S. aureus* strains with the MIC spanning from 8 to 128 μg/mL. An interesting note concerns the activity of these four compounds against *S. epidermidis* where, in general, they showed the best efficacy by reaching MIC values from 1 to 16 μg/mL. In particular, compound **2b** showed bacteriostatic effects against all three tested Gram-positive bacteria and, in opposition, compound **2e** was observed to have a bactericidal effect against these three strains. On the other hand, **2a** and **2f** showed different effects among the bacteria tested with a bacteriostatic effect against *S. aureus* and a bactericidal effect for the other two strains by **2a**. Conversely, **2f** provided a bactericidal effect against *E. faecalis* and a bacteriostatic effect for the other two strains. Taking into consideration the results of the MIC on the *S. epidermidis* strain, it was decided to evaluate our derivatives **2a**, **2b**, **2e**, and **2f** under irradiation with a 652 nm laser for 47 min in order to increase their antimicrobial effects against this strain. With the exception of compound **2e**, where the irradiation seemed not have any impact on antibacterial activity, the other derivatives showed an increase in activity of an order of magnitude, and **2b** reduced the growth of irradiated *S. epidermidis* by 2 logs (Table 3).

Finally, to evaluate the MBC values, the compounds were also tested at concentrations up to 8 × MIC to assess their bacteriostatic or bactericidal effects (Table 2).

**Figure 3:**
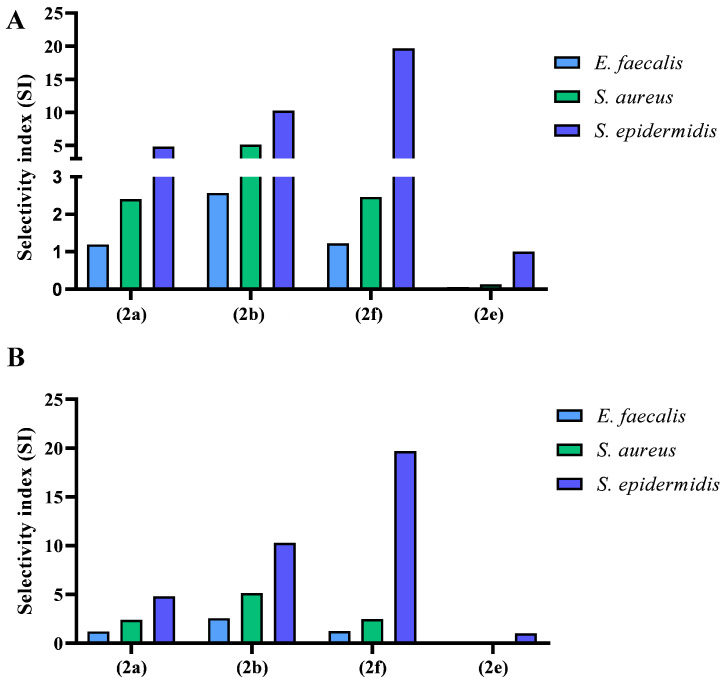
Selectivity indexes (SI) of **2a**, **2b**, **2e**, and **2f**, which displayed antibacterial activity against *E. faecalis*, *S. aureus*, and *S. epidermidis*. The SI was calculated as the ratio between cytotoxicity in μg/mL and the MIC value in μg/mL against each bacterium: (**A**) without irradiation, (**B**) with irradiation.

Since our compounds were able to influence bacterial growth, their cytotoxicity against human fibroblasts (MRC-5 cell line) was studied, and the results are reported in Table 2. Compounds **2c** and **2d**, which failed to produce any antimicrobial effect, were also not toxic to MRC-5 cells up to 256 µg/mL. On the other hand, compound **2e**, which showed the best activity against *S. epidermidis* with a MIC of 1–4 µg/mL, had in high cytotoxicity to human fibroblasts with an IC_50_ of 2 µg/mL. In contrast, compound **2a** was 19-fold less toxic than **2e** was with an IC_50_ of 38.5 µg/mL. Notably, **2b** and **2e** showed low cytotoxicity against the human fibroblast cell line tested here, with IC_50_ values of 82.2 and 78.8 µg/mL, respectively, far from the MIC values. Moreover, both the high MIC value and high cytotoxicity, as well as its lack of selectivity for bacterial strains, made compound **2e** unsuitable for further developments (Figure 3). In contrast, **2f** showed the highest SI against *S. epidermidis*. *S. epidermidis* is a part of the normal microbiota and is not usually pathogenic, but immunocompromised patients can develop hospital-acquired infections. These infections are generally related to the use of catheters or other surgical devices where drug-resistant biofilms form.

**Figure 4:**
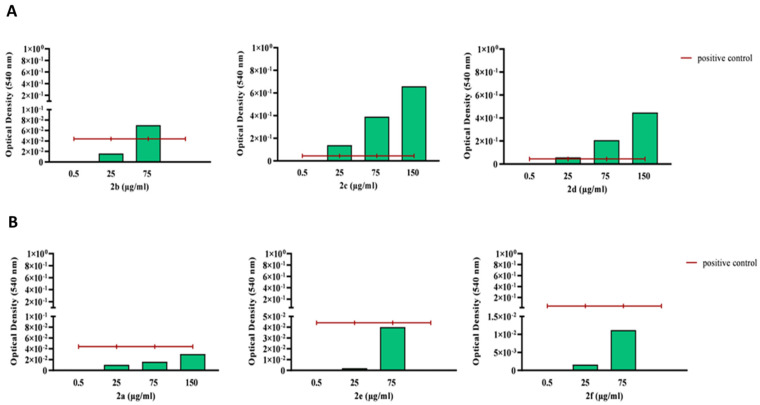
Optical density measured at 540 nm upon product incubation (0.5–150 µg/mL) with human blood cells, with compounds displaying cytotoxicity (upper panel, (**A**)) and non-cytotoxic compounds (lower panel, (**B**)). The hemolysis assay was performed according to an established protocol, and optical density was measured at 540 nm. The blank was subtracted, and the obtained values were normalized to those of the positive control (0.01% Tween), displayed as a red line.

Subsequently, the hemolysis activity of all the compounds was studied. The analysis showed that in comparison to a positive control (cells incubated with 0.01% Tween 20), products **2a**, **2e**, and **2f** were not cytotoxic to human red blood cells. On the other hand, products **2b**, **2c**, and **2d** showed significant cytotoxicity, especially at higher concentrations. Compound **2b** only seemed to be cytotoxic above the concentration of 75 μg/mL (Figure 4), a concentration much higher than its MIC value.

## 3. Materials and Methods

### 3.1. General

Anhydrous solvents and all reagents were purchased from Sigma-Aldrich, VWR, and TCI. All reactions involving air- or moisture-sensitive compounds were performed under a nitrogen atmosphere. Nuclear magnetic resonance (^1^H NMR, ^13^C NMR) spectra were recorded using a Bruker Advance III 400 MHz spectrometer in DMSO-*d*_6_ or CDCl_3_. Chemical shifts are reported in parts per million (ppm), and the coupling constants (*J*) are expressed in hertz (Hz). Splitting patterns are designated as follows: s, singlet; d, doublet; t, triplet; m, multiplet; br, broad; ap, apparent; dd, doublet of doublets; ls, line separation. The assignment of exchangeable protons (N*H*) was confirmed by the addition of D_2_O. Analytical thin-layer chromatography (TLC) was carried out on Merck silica gel F-254 plates. Flash chromatography purifications were performed on Merck silica gel 60 (230–400 mesh ASTM) as the stationary phase, and ethyl acetate, *n*-hexane, acetonitrile, and methanol were used as eluents. The solvents used in MS measurements were acetone, acetonitrile (Chromasolv grade), purchased from Sigma-Aldrich (Milan, Italy), and mQ water 18 MΩ, obtained from Millipore’s Simplicity system (Milan, Italy). Mass spectra were obtained using a Varian 1200L triple quadrupole system (Palo Alto, CA, USA) equipped with an electrospray source (ESI) operating in both positive and negative ions. UV–Vis spectra in different solvents were recorded with a Shimadzu UV-2600 spectrometer. Fluorescence spectra on solutions were measured at 20 °C with a Jasco FP-8300 spectrofluorometer equipped with a 450 W Xenon arc lamp. Stock solutions of analytes were prepared in acetone at 1.0 mg mL^−1^ and stored at 4 °C. Working solutions of each analyte were freshly prepared by diluting stock solutions in a mixture of mQ H_2_O/ACN 1/1 (*v*/*v*) up to a concentration of 1.0 μg mL^−1^. The mass spectra of each analyte were acquired by introducing the working solution via a syringe pump at 1.00 mL min^−1^. Raw data were collected and processed by Varian Workstation software, version 6.8. All compounds reported here were >95% pure.

### 3.2. General Procedure for the Synthesis of Compounds ***2a***,***c***,***e***

*N,N*-Diisopropylethylamine (DIPEA) (0.35 mmol, 1.5 equiv) was added to a solution of **1a** (0.26 mmol, 1.1 equiv) in dry acetonitrile (10 mL) under magnetic stirring at room temperature. Then, the electrophile (IR-780 iodide, IR-820, or IR-813 perchlorate: 0.24 mmol, 1.0 equiv) was added, and the mixture was refluxed (90 °C) for 24 h under continuous magnetic stirring. Afterwards, the solvent was removed under a vacuum, and the crude material was purified by flash chromatography on a silica gel to afford **2a**,**c**,**e**.

### 3.3. General Procedure for the Synthesis of Compounds ***2b***,***d***,***f***

*N,N*-Diisopropylethylamine (DIPEA) (0.71 mmol, 3.0 equiv) was added to a solution of 1b (0.26 mmol, 1.1 equiv) in dry acetonitrile (10 mL) under magnetic stirring at room temperature. Then, the electrophile (IR-780 iodide, IR-820, or IR-813 perchlorate: 0.24 mmol, 1.0 equiv) was added, and the mixture was refluxed (90 °C) for 24 h under continuous magnetic stirring. Afterwards, the solvent was removed under a vacuum, and the crude material was purified by flash chromatography on a silica gel to afford **2a**,**c**,**e**.

### 3.4. Synthesis of Compound ***2a***

Following the general procedure, IR-780 iodide (100 mg, 0.15 mmol) and **1a** (33 mg, 0.17 mmol) gave, after purification by flash column chromatography (CHCl_3_:MeOH 15:1), 2a as a blue solid (67 mg, 54%). ^1^H NMR (CD_3_OD, 400 MHz) *δ* (ppm): 1.02 (6H, t, *J* = 7.4 Hz), 1.59 (12H, s), 1.72–1.84 (6H, m), 2.46 (4H, bt, *J* = 6.1 Hz), 3.19 (2H, bt, *J* = 6.3 Hz), 3.91 (4H, bt, *J* = 7.2 Hz), 4.13 (2H, bt, *J* = 6.5 Hz), 5.81 (2H, d, *J* = 13.0 Hz), 7.06–7.11 (4H, m), 7.30 (2H, ap t, ls = 7.7 Hz), 7.37 (2H, ap d, ls = 7.7 Hz), 7.45 (2H, ap d, ls = 8.1 Hz), 7.68 (2H, d, *J* = 13.0 Hz), 7.86 (2H, ap d, ls = 8.1 Hz). ^13^C NMR (CD_3_OD, 100 MHz) *δ* (ppm): 11.0, 20.4, 21.9, 25.3, 28.4, 37.1, 44.8, 50.6, 52.6, 95.2, 109.5, 120.7, 122.3, 123.3, 126.9, 128.6, 129.6, 139.1, 140.6, 142.8, 143.2, 143.8, 168.8, 169.7. MS (ESI, positive): 703.3, M^+^.

### 3.5. Synthesis of Compound ***2b***

Following the general procedure, IR-780 iodide (200 mg, 0.30 mmol) and **1b** (123 mg, 0.33 mmol) gave, after purification by flash column chromatography (CHCl_3_:MeOH 15:1), **2b** as a blue solid (139 mg, 52%). ^1^H NMR (CD_3_OD, 400 MHz) *δ* (ppm): 1.01 (6H, t, *J* = 7.4 Hz), 1.65 (12H, s), 1.77–1.84 (6H, m), 1.77–2.00 (6H, m), 2.53 (4H, t, *J* = 5.6 Hz), 3.41 (2H, t, *J* = 5.9 Hz), 3.82–3.90 (6H, m), 5.80 (2H, d, *J* = 12.9 Hz), 7.04 (2H, ap d, ls = 7.8 Hz), 7.07 (2H, ap d, ls = 7.6 Hz), 7.27 (2H, ap d, ls = 7.8 Hz), 7.31 (2H, ap d, ls = 7.6 Hz), 7.58 (2H, ap d, ls = 8.2 Hz), 7.75 (2H, ap d, ls = 8.2 Hz), 7.78 (2H, d, *J* = 12.9 Hz). ^13^C NMR (CD_3_OD, 100 MHz) *δ* (ppm): 11.0, 20.3, 22.0, 25.5, 28.4, 30.0, 32.5, 36.7, 44.7, 94.9, 109.4, 118.0, 120.7, 122.2, 123.1, 127.5, 128.6, 136.8, 138.9, 140.5, 143.8, 144.2, 157.0, 168.4, 170.2. MS (ESI, positive): 761.4, M^+^.

### 3.6. Synthesis of Compound ***2c***

Following the general procedure, IR-820 (200 mg, 0.24 mmol) and **1a** (52 mg, 0.26 mmol) gave, after purification by flash column chromatography (CHCl_3_:MeOH 5:1), **2c** as a blue solid (158 mg, 65%). ^1^H NMR (CD_3_OD, 400 MHz) *δ* (ppm): 1.61–1.70 (2H, m), 1.81 (12H, s), 1.88–2.00 (8H, m), 2.43 (4H, ap bs), 2.89 (4H, ap bs), 3.18 (2H, ap bs), 4.00 (4H, ap bs), 4.13 (2H, ap bs), 4.59 (2H, bs), 5.78 (2H, d, *J* = 12.8 Hz), 7.31 (2H, ap t, ls = 7.5 Hz), 7.36 (2H, ap d, ls = 8.6 Hz), 7.45 (2H, ap d, ls = 7.9 Hz), 7.50 (2H, ap t, ls = 7.9 Hz), 7.73 (2H, d, *J* = 12.8 Hz), 7.80–7.88 (4H, m), 7.89 (2H, ap d, ls = 7.9 Hz), 8.09 (2H, ap d, ls = 8.6 Hz). ^13^C NMR (CD_3_OD, 100 MHz) *δ* (ppm): 21.9, 22.9, 25.4, 26.3, 27.9, 37.2, 43.3, 50.0, 50.7, 51.3, 94.9, 110.7, 122.3, 124.1, 126.9, 127.6, 129.0, 129.7, 130.2, 130.5, 131.7, 131.8, 138.4, 141.0, 142.7, 143.5, 169.3, 170.1. MS (ESI, negative): 989.4, M^─^.

### 3.7. Synthesis of Compound ***2d***

Following the general procedure, IR-820 (200 mg, 0.24 mmol) and **1b** (96 mg, 0.26 mmol) gave, after purification by flash column chromatography (CHCl_3_:MeOH 3:1), **2d** as a blue solid (127 mg, 50%). ^1^H NMR (CD_3_OD, 400 MHz) *δ* (ppm): 1.81–2.02 (10H, m), 1.92 (12H, s), 2.55 (4H, ap bs), 2.88 (4H, ap bs), 3.40 (2H, ap bs), 3.87 (2H, ap bs), 4.05 (4H, ap bs), 5.83 (2H, d, *J* = 12.5 Hz), 7.34 (2H, ap t, ls = 7.5 Hz), 7.41 (2H, ap d, ls = 8.8 Hz), 7.49 (2H, ap d, ls = 7.5 Hz), 7.65–7.67 (2H, m), 7.75 (2H, d, ls = 8.8 Hz), 7.83–7.91 (6H, m), 8.08 (2H, ap d, ls = 8.5 Hz). MS (ESI, negative): 1047.2, M^─.^

### 3.8. Synthesis of Compound ***2e***

Following the general procedure, IR-813 perchlorate (250 mg, 0.37 mmol) and **1a** (81 mg, 0.4 mmol) gave, after purification by flash column chromatography (CHCl_3_:MeOH 15:1), **2e** as a blue solid (104 mg, 33%). ^1^H NMR (CD_3_OD, 400 MHz) *δ* (ppm): 1.75–1.80 (2H, m), 1.90 (12H, s), 2.51–2.54 (4H, m), 3.25 (2H, t, *J* = 6.4 Hz), 3.55 (6H, s), 4.19 (2H, t, *J* = 6.4 Hz), 5.81 (2H, d, *J* = 13.2 Hz), 7.38 (2H, ap t, ls = 7.4 Hz), 7.44 (2H, ap d, ls = 8.8 Hz), 7.49 (2H, ap d, ls = 8.0 Hz), 7.55–7.59 (2H, m), 7.82 (2H, d, *J* = 13.2 Hz), 7.89–7.95 (6H, m), 8.17 (2H, ap d, ls = 8.5 Hz). ^13^C NMR (CD_3_OD, 100 MHz) *δ* (ppm): 22.0, 22.3, 27.0, 27.7, 37.1, 50.0, 50.6, 94.8, 110.4, 122.3, 124.1, 126.9, 127.6, 129.6, 130.2, 130.5, 131.8, 138.7, 141.6, 143.4, 169.4, 171.2. MS (ESI, positive): 747.5, M^+^.

### 3.9. Synthesis of Compound ***2f***

Following the general procedure, IR-813 perchlorate (250 mg, 0.37 mmol) and **1b** (150 mg, 0.40 mmol) gave, after purification by flash column chromatography (CHCl_3_:MeOH 15:1), **2f** as a blue solid (112 mg, 34%). ^1^H NMR (CD_3_OD, 400 MHz) *δ* (ppm): 1.85–1.90 (2H, m), 1.96 (12H, s), 2.59 (4H, t, *J* = 6.2 Hz), 3.44–3.48 (2H, m), 3.54 (6H, s), 3.90 (2H, t, *J* = 6.2 Hz), 5.80 (2H, d, *J* = 13.1 Hz), 7.36 (2H, ap t, ls = 7.4 Hz), 7.42 (2H, ap d, ls = 8.8 Hz), 7.51 (2H, ap d, ls = 7.4 Hz), 7.60 (2H, ap d, ls = 8.8 Hz), 7.75 (2H, ap d, ls = 8.8 Hz), 7.89–7.96 (6H, m), 8.10 (2H, ap d, ls = 8.5 Hz). MS (ESI, positive): 805.5, M^+^.

### 3.10. Carbonic Anhydrase Inhibition

An Applied Photophysics stopped-flow instrument was used to assay CA-catalyzed CO_2_ hydration activity [19]. Phenol red (at a concentration of 0.2 mM) was used as an indicator, working at the absorbance maximum of 557 nm, with 20 mM Hepes (pH 7.4 for α-class and pH 8.4 for β- and γ-classes) as a buffer and 20 mM Na_2_SO_4_ (to maintain constant the ionic strength), following the initial rates of the CA-catalyzed CO_2_ hydration reaction for a period of 10–100 s. The CO_2_ concentrations ranged from 1.7 to 17 mM for the determination of the kinetic parameters and inhibition constants [20]. Enzyme concentrations ranged between 5 and 12 nM. For each inhibitor, at least six traces of the initial 5–10% of the reaction were used to determine the initial velocity. The uncatalyzed rates were determined in the same manner and subtracted from the total observed rates. Stock solutions of the inhibitor (0.1 mM) were prepared in distilled–deionized water, and dilutions up to 0.01 nM were done thereafter with the assay buffer. Inhibitor and enzyme solutions were preincubated together for 15 min at room temperature prior to the assay to allow for the formation of the E–I complex. The inhibition constants were obtained by non-linear least-squares methods using PRISM 3 and the Cheng–Prusoff equation as reported earlier and represent the mean from at least three different determinations. All CA isoforms were recombinant proteins obtained in house, as reported earlier [12,21,22].

### 3.11. Bacteria Culturing Conditions and Antibacterial Properties Assays

Derivatives **2a**–**f** were dissolved in DMSO and further diluted with distilled water to the required final concentration. The microorganisms (*E. faecalis*, *S. aureus*, and *S. epidermidis* reported in Table 4) were cultured overnight at 35 °C in Mueller Hinton Broth 2, Cation-Adjusted (BD, BD 212322, New York, NY, USA) and adjusted to 0.5 McFarland, according to standardized guidelines NF EN ISO 20776-1 [23]. The minimal inhibitory concentration (MIC) test was performed according to the standardized guidelines NF EN ISO 20776-1 [23]. Once the MIC was obtained, the minimal bactericidal concentration (MBC) test was performed. The cells were plated with the compounds at MIC, 2 × MIC, 4 × MIC, and 8 × MIC, and incubated overnight at 35 °C on Mueller Hinton Agar (BD, 225250, New York, NY, USA).

### 3.12. Photoactivity Properties Assay

*S. epidermidis* was incubated with the products at an average concentration of 64 μg/mL for **2c** and **2d**, and the rest of products were incubated at their MIC values. Upon incubation of 1.5–2 h with the products, the samples were irradiated for 47 min with a 652 nm laser (to obtain 1.70 W, 30 J/cm^2^). Upon irradiation, the cells were plated and incubated overnight at 35 °C on Mueller Hinton Agar (BD, 225250, New York, NY, USA).

### 3.13. Cytotoxicity

The cytotoxicity of the products was tested on MRC-5 cells (lung fibroblasts, ATCC CCL-171) with a seeding concentration of 10,000 cells/well. The products were diluted with a 2% FSC MEM medium (Sigma, M4655, Missouri, MO, USA) to concentrations ranging from 1 to 256 μg/mL and incubated with 80% confluent cells for 24 h. The following day, a 3-[4,5-dimethylthiazol-2-yl]-2,5-diphenyl tetrazolium bromide (MTT) assay was performed, and the optical density of the samples was measured at 540 nm.

### 3.14. Hemolysis

Blood collected at CHU Nancy was washed three times with PBS, and 10^7^ cells/mL were incubated for 30 min at 37 °C with the newly synthesized compounds. The derivatives were previously diluted to the range from 0.5 to 75/150 μg/mL with PBS. As a positive control, cells were resuspended with 0.01% polysorbate 20 (Tween 20). Tween 20 has often been used as a reference compound for cell lysis [24]. Upon incubation, the samples were centrifuged for 5 min at 800× *g*, and the supernatant was collected for optical density reading at 540 nm.

## 4. Conclusions

Photoactivatable compounds (**2a**–**f**) were designed to display CA inhibition, synthesized for the treatment of microbial infections, and assayed against several isoforms of bacterial carbonic anhydrase, displaying good selectivity compared with that to off-target human CAs I and II. In particular, derivatives **2a**, **2b**, and **2f** showed moderate to good antibacterial activity against Gram-positive bacteria such as *E. faecalis*, *S. aureus*, and *S. epidermidis*, as also recently demonstrated in Gram-negative bacteria by other chemical scaffolds [25,26]. When these compounds were irradiated with a 652 nm laser, except for compound **2e**, at least 1 log of growth reduction was observed for *S. epidermidis* and, notably, 2 log of growth reduction with compound **2b**. A slight growth inhibition could indicate the boost of activity upon irradiation, which suggests that the synthesized molecules were indeed photoactive. Certain solvents and media components could quench the production of singlet oxygen and reactive oxygen species, which could explain reduced photoactivation in some molecules. Additionally, some molecules absorb beyond selected irradiation wavelengths, which could also explain the limited photoresponse. According to the above-described data, our molecules showed great potential, despite some modifications that might be needed to reduce cytotoxicity and increase the selectivity index. An investigation into the mechanism of action might contribute to further molecule development, which could enhance existing potential and lead to the products’ application in antibacterial photodynamic therapy.

## Figures and Tables

**Table 1:** Inhibition data of human and bacterial CA isoforms, with compounds **2a**–**f** and **AAZ** in a stopped-flow CO_2_ hydrase assay.

*K*_I_ (nM) ^1^								
**Cmpd**	**hCA I**	**hCA II**	**MscCA**	***E. coli* β**	***E. coli* γ**	**PsCA1**	**PsCA3**	**SmuCA**
**2a**	410.0	163.3	62.5	54.8	120.3	60.4	89.4	102.4
**2b**	957.1	587.9	55.8	69.4	93.5	46.9	48.1	73.3
**2c**	529.0	91.0	59.3	60.2	117.1	64.8	76.8	99.5
**2d**	923.3	182.7	68.3	69.8	111.1	88.7	81.0	92.7
**2e**	860.8	159.7	67.2	74.1	109.8	71.1	77.7	103.5
**2f**	3899	624.7	74.5	64.9	123.8	58.3	94.5	85.2
**AAZ**	250.0	12.1	628	227	248	37	75.9	344

^1^ Mean from 3 different assays with a stopped-flow technique (errors were in the range of ±5–10% of the reported values). *K*_I_ = constant of inhibition.

**Table 2:** Values of MIC, MBC, and cytotoxicity against MRC-5 cell line of compounds **2a–f**.

*E. faecalis*			*S. aureus*		*S. epidermidis*		MRC-5
Cmpd	MIC (µg/mL)	MBC	MIC (µg/mL)	MBC	MIC (µg/mL)	MBC	IC_50_
**2a**	16–64	bactericidal	8–32	bacteriostatic	4–16	bactericidal	38.5 µg/mL
**2c**	>256	>256	>256	>256	>256	>256	>256 µg/mL
**2b**	16–64	bacteriostatic	8–32	bacteriostatic	4–16	bacteriostatic	82.2 µg/mL
**2f**	32–128	bactericidal	16–64	bacteriostatic	2–8	bacteriostatic	78.8 µg/mL
**2d**	>256	>256	>256	>256	>256	>256	>256 µg/mL
**2e**	16–64	bactericidal	8–32	bactericidal	1–4	bactericidal	2 µg/mL

**Table 3:** Growth reduction of *S. epidermis* treated with laser-activated (652 nm) compounds **2a**–**f**.

*S. epidermidis*		
Compound	MBC	Growth Reduction
**2a**	bactericidal	1 log reduction
**2c**	>256	1 log reduction
**2b**	bacteriostatic	2 log reduction
**2f**	bacteriostatic	1 log reduction
**2d**	>256	1 log reduction
**2e**	bactericidal	0 log reduction

**Table 4:** Origin of experimental microorganisms.

Genus	Species	Origin	Year
*Enterococcus*	*faecalis*	Clinical isolate, CHU Nancy	2021
*Staphylococcus*	*aureus*	ATCC 29213,	2020/21
*Staphylococcus*	*epidermidis*	ATCC 14990,	2020

## Data Availability

Not applicable.

## Supplementary Materials

The supporting information can be downloaded at: https://www.mdpi.com/article/10.3390/ijms24119610/s1.
