# Supplementary material for: Photoactivatable Heptamethine-Based Carbonic Anhydrase Inhibitors Leading to New Anti-Antibacterial Agents
Source: Int J Mol Sci. 2023 Jun 1;24(11):9610. doi: 10.3390/ijms24119610 (PMC10254030; doi:10.3390/ijms24119610)

SUPPORTING INFORMATION

Photo-activatable heptamethine-based carbonic anhydrase inhibitors leading to new anti-antibacterial agents

Simone Carradori,[1] Andrea Angeli,[2]* Patrick S. Sfragano,[3] Xheila Yzeiri,[3,4,5] Massimo Calamante,[3,4] Damiano Tanini,[3]* Antonella Capperucci,[3] Hannah Kunstek,[6,7] Mihayl Varbanov,[7,8] Clemente Capasso,[9] Claudiu T. Supuran[2]

1 Department of Pharmacy, "G. d'Annunzio" University of Chieti-Pescara, Chieti, Italy

2 NEUROFARBA Department, Sezione di Scienze Farmaceutiche, University of Florence, Via Ugo Schiff 6, 50019 Sesto Fiorentino, Florence, Italy

3 Department of Chemistry "Ugo Schiff", University of Florence, Via Della Lastruccia 3-13, I-50019, Sesto Fiorentino, Italy

4 CNR-Institute of Chemistry of Organometallic Compounds (CNR-ICCOM), Via Madonna del Piano 10, 50019 Sesto Fiorentino, Italy

5 Department of Biotechnology, Chemistry and Pharmacy, University of Siena, Siena 53100, Italy

6 Graz University of Technology, 8010 Graz, Austria

7 L2CM, Université de Lorraine, Centre National de la Recherche Scientifique (CNRS), 54000 Nancy, France

8 Laboratoire de Virologie, Centres Hospitaliers Régionaux Universitaires (CHRU) de Nancy Brabois, 54500 Vandœuvre-lès-Nancy, France

9 Department of Biology, Agriculture and Food Sciences, National Research Council (CNR), Institute of Biosciences and Bioresources, 80131 Naples, Italy

INDEX

Absorption (red line) and emission spectra ***S2-S4***

NMR Spectra ***S5-S8***

Carbonic Anhydrase activity ***S9***

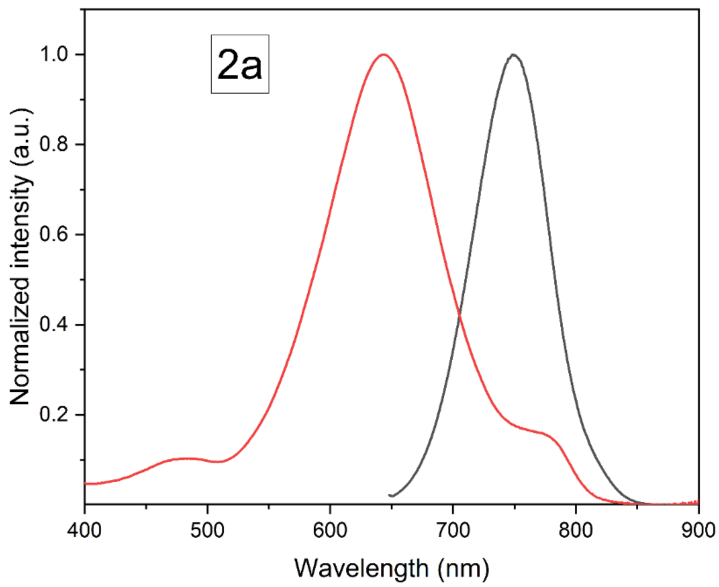

Figure S1. Absorption (red line) and emission spectra (black line) of compound **2a** in methanol solution (conc.: 2.4·10^{-6} M) (excitation wavelength 643 nm).

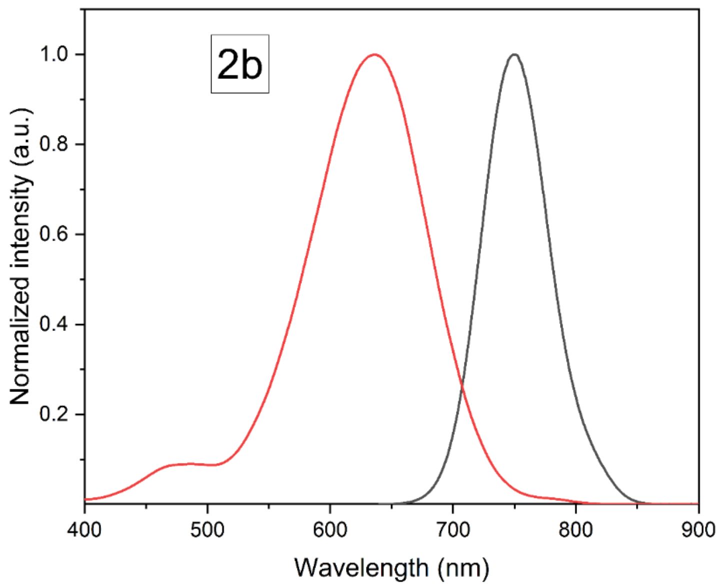

Figure S2. Absorption (red line) and emission spectra (black line) of compound **2b** in methanol solution (conc.: 3.1·10^{-6} M) (excitation wavelength 636 nm).

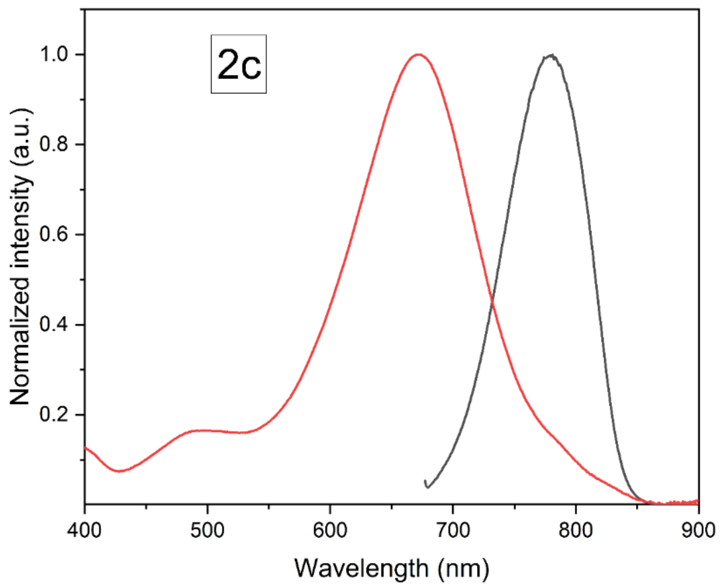

Figure S3. Absorption (red line) and emission spectra (black line) of compound **2c** in methanol solution (conc.: 2.3·10^{-6} M) (excitation wavelength 672 nm).

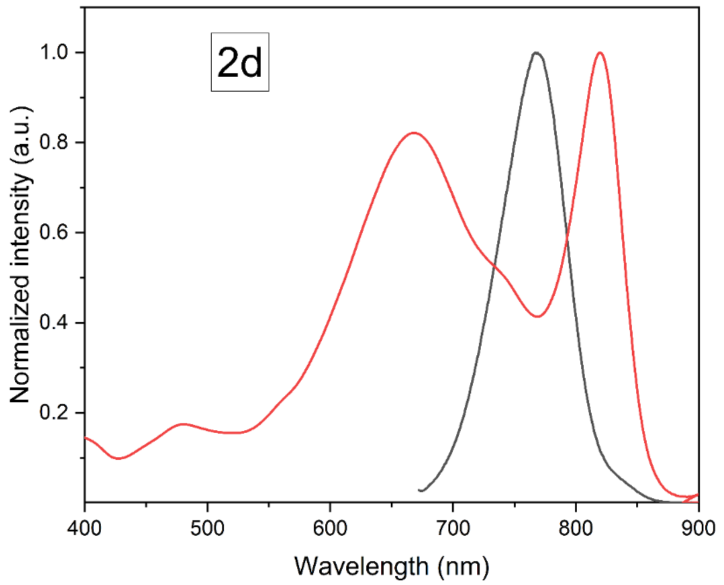

Figure S4. Absorption (red line) and emission spectra (black line) of compound **2d** in methanol solution (conc.: 6.2·10^{-6} M) (excitation wavelength 668 nm).

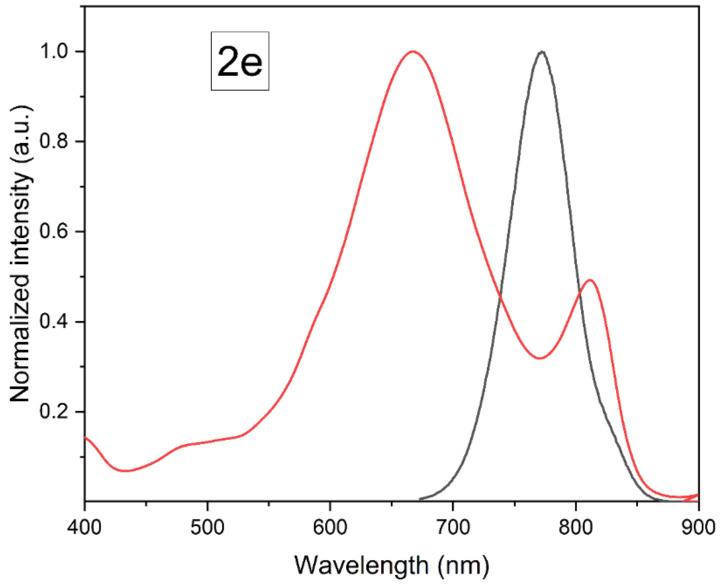

Figure S5. Absorption (red line) and emission spectra (black line) of compound **2e** in methanol solution (conc.: $4.0 \cdot 10^{-6}$ M) (excitation wavelength 667 nm).

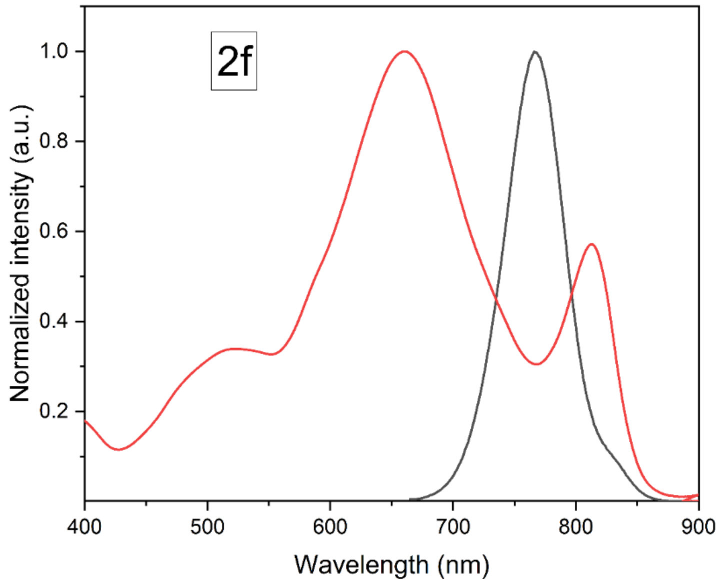

Figure S6. Absorption (red line) and emission spectra (black line) of compound **2f** in methanol solution (conc.: $5.3 \cdot 10^{-6}$ M) (excitation wavelength 661 nm).

NMR SPECTRA

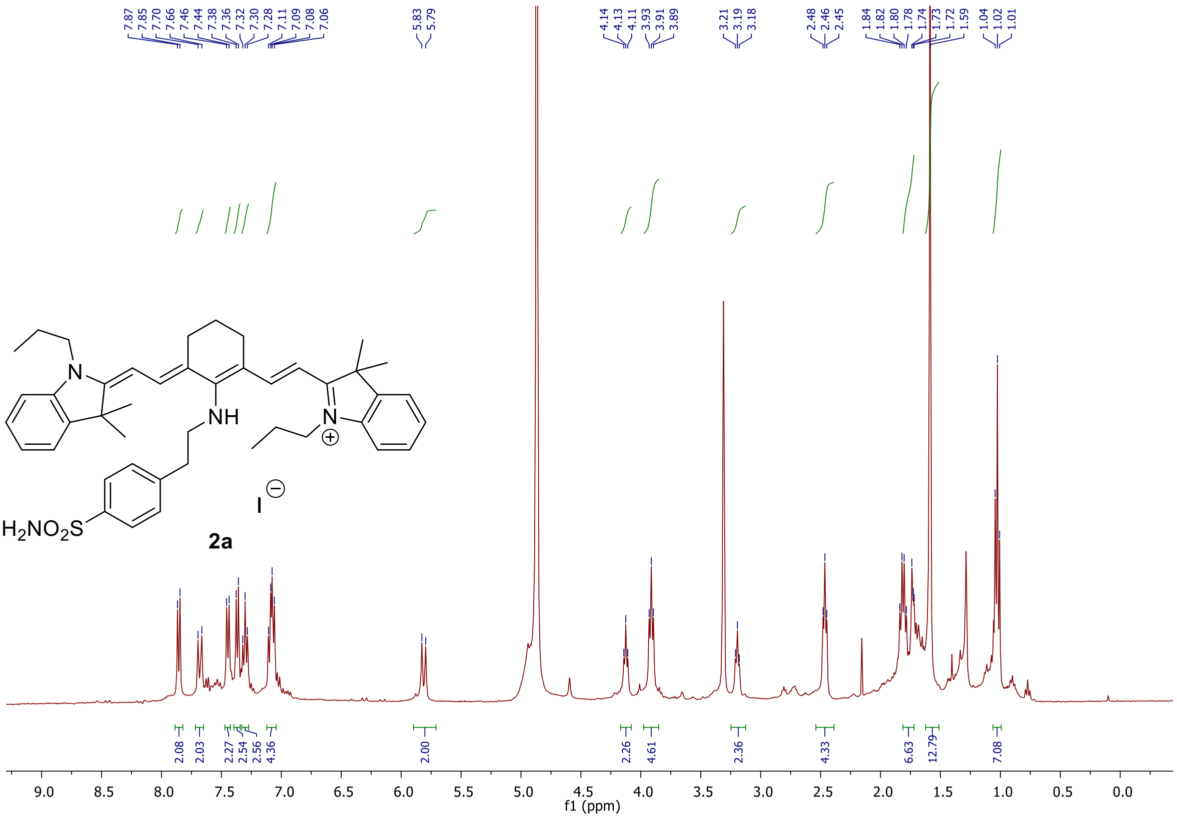

^{1}H NMR spectrum (CD_3OD) of compound **2a** – 400 MHz

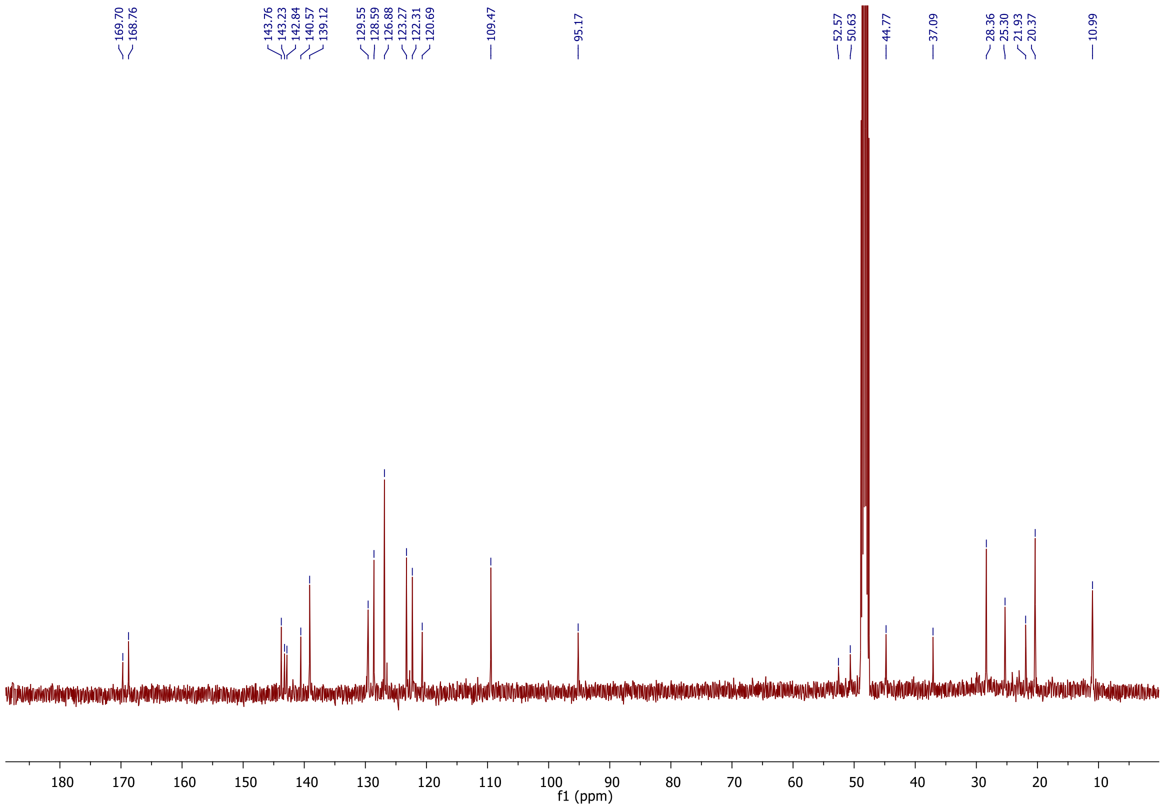

^{13}C NMR spectrum (CD_3OD) of compound **2a** – 100 MHz

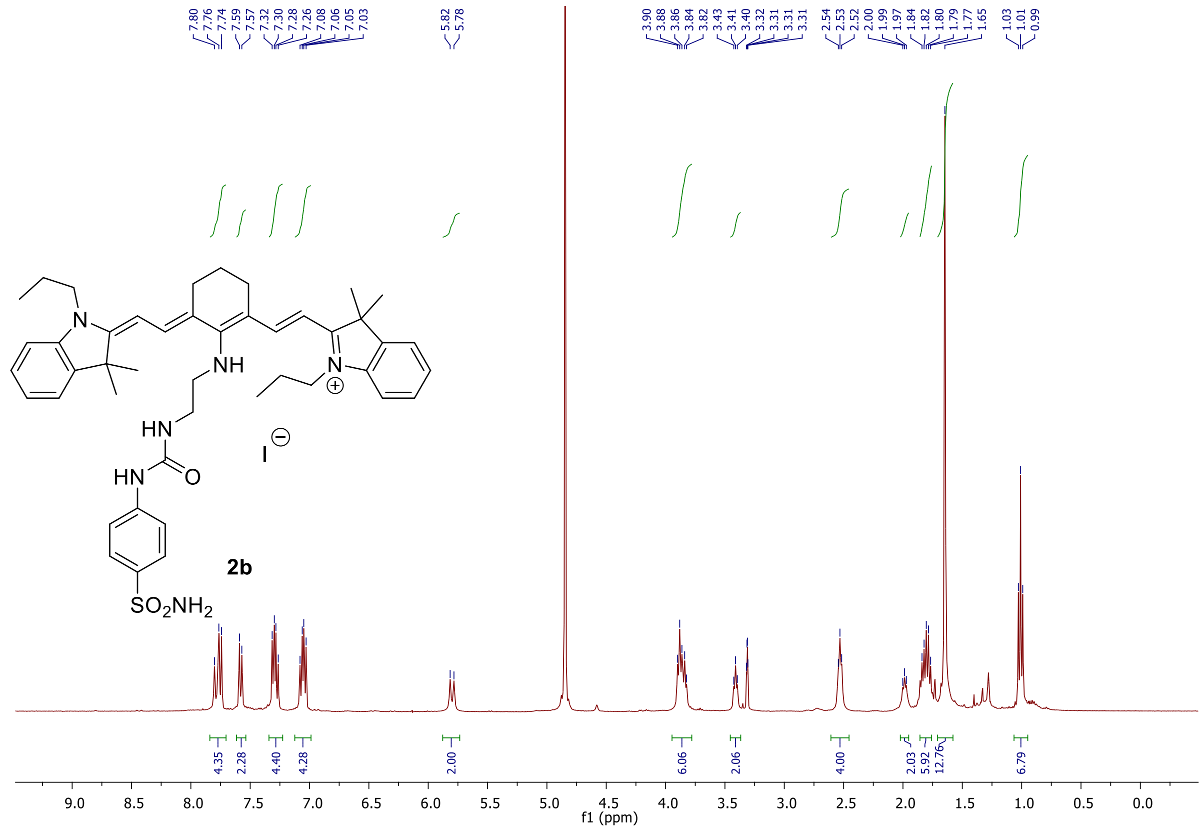

^{1}H NMR spectrum (CD_3OD) of compound **2b** – 400 MHz

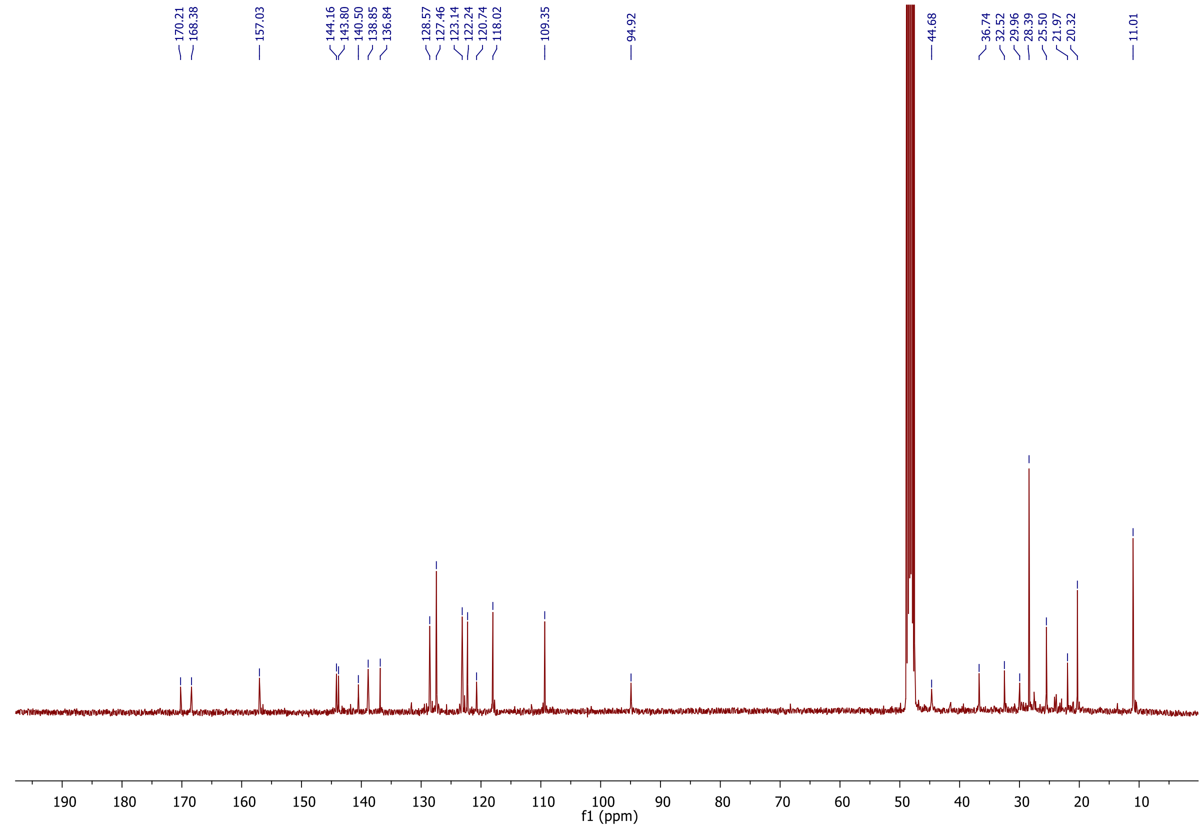

^{13}C NMR spectrum (CD_3OD) of compound **2b** – 100 MHz

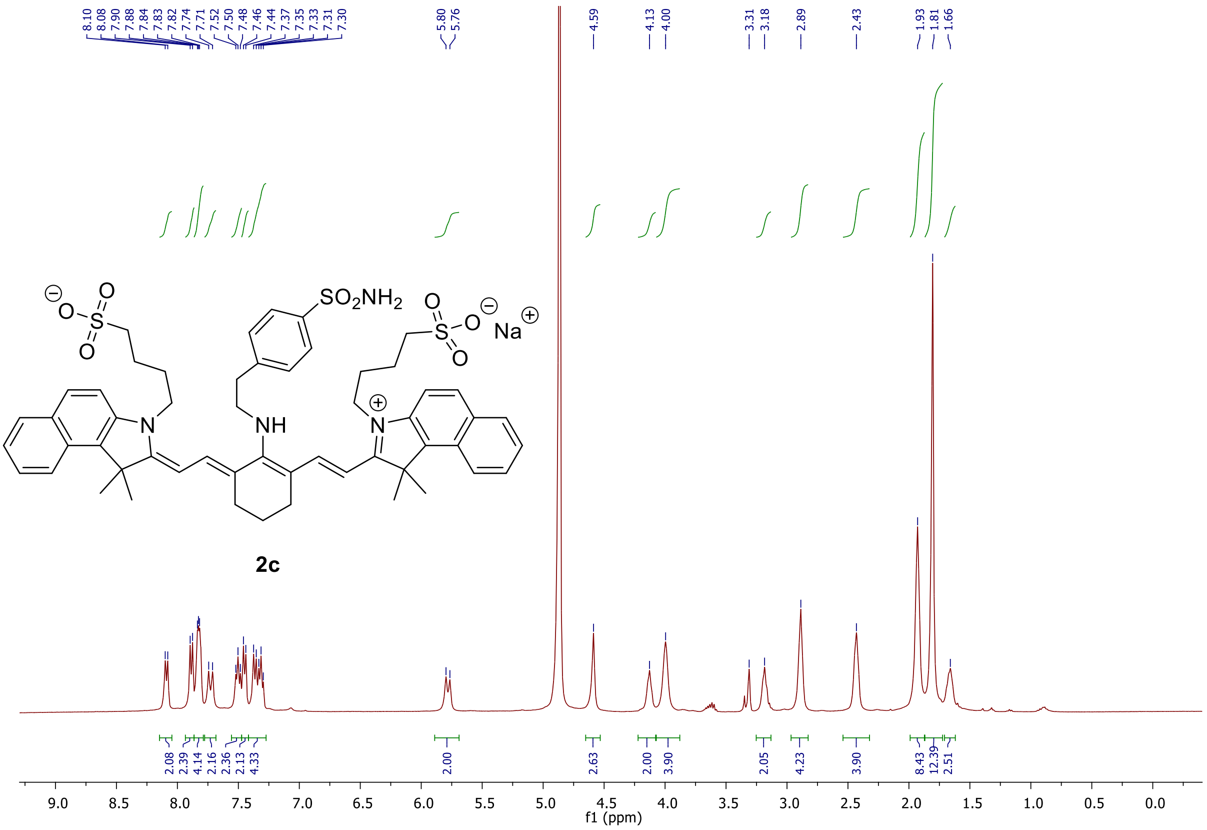

^{1}H NMR spectrum (CD_3OD) of compound **2c** – 400 MHz

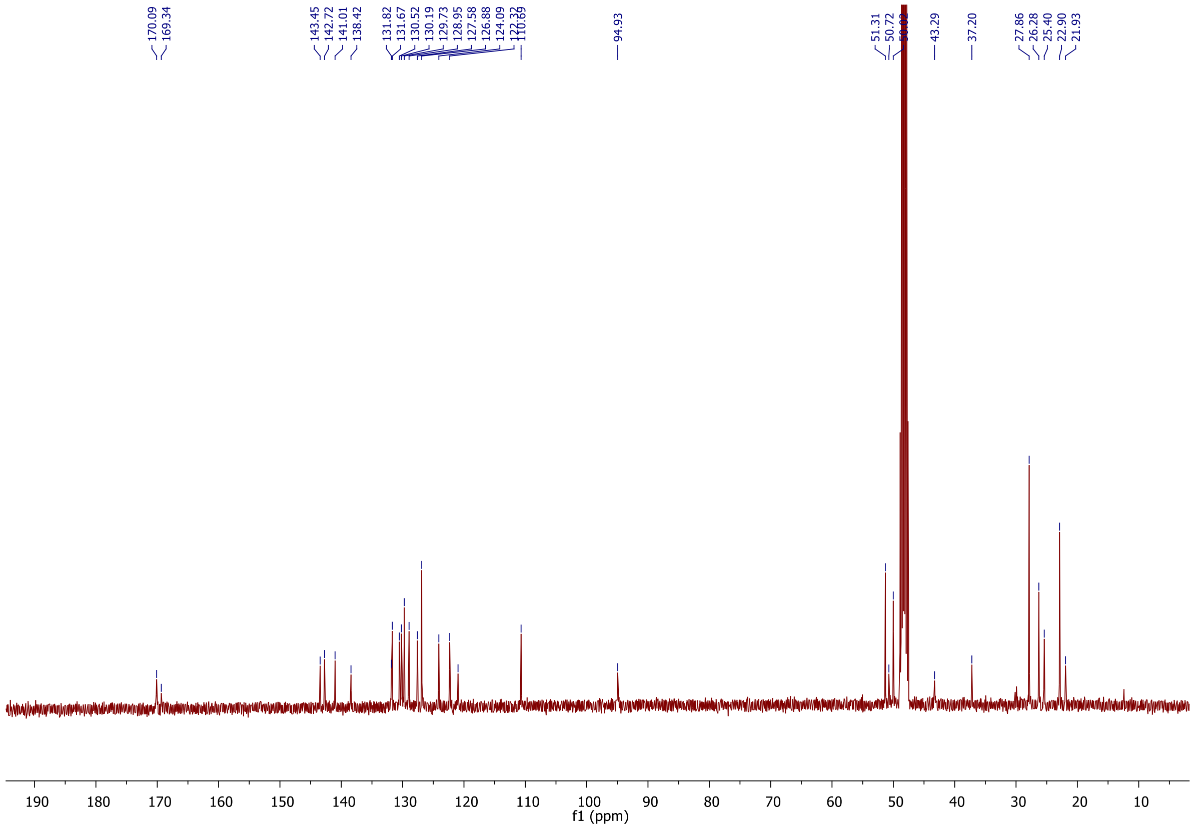

^{13}C NMR spectrum (CD_3OD) of compound **2c** – 100 MHz

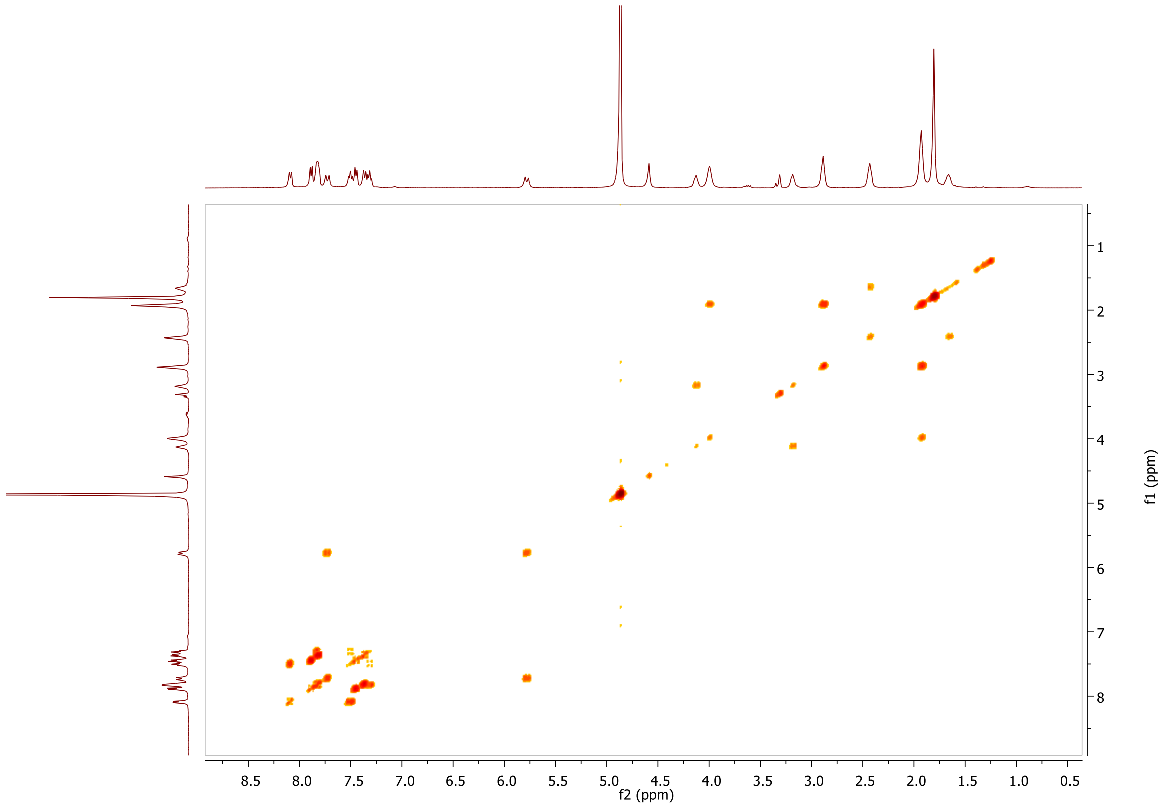

^1H-^1H COSY spectrum (CD_3OD) of compound **2c** – 400 MHz

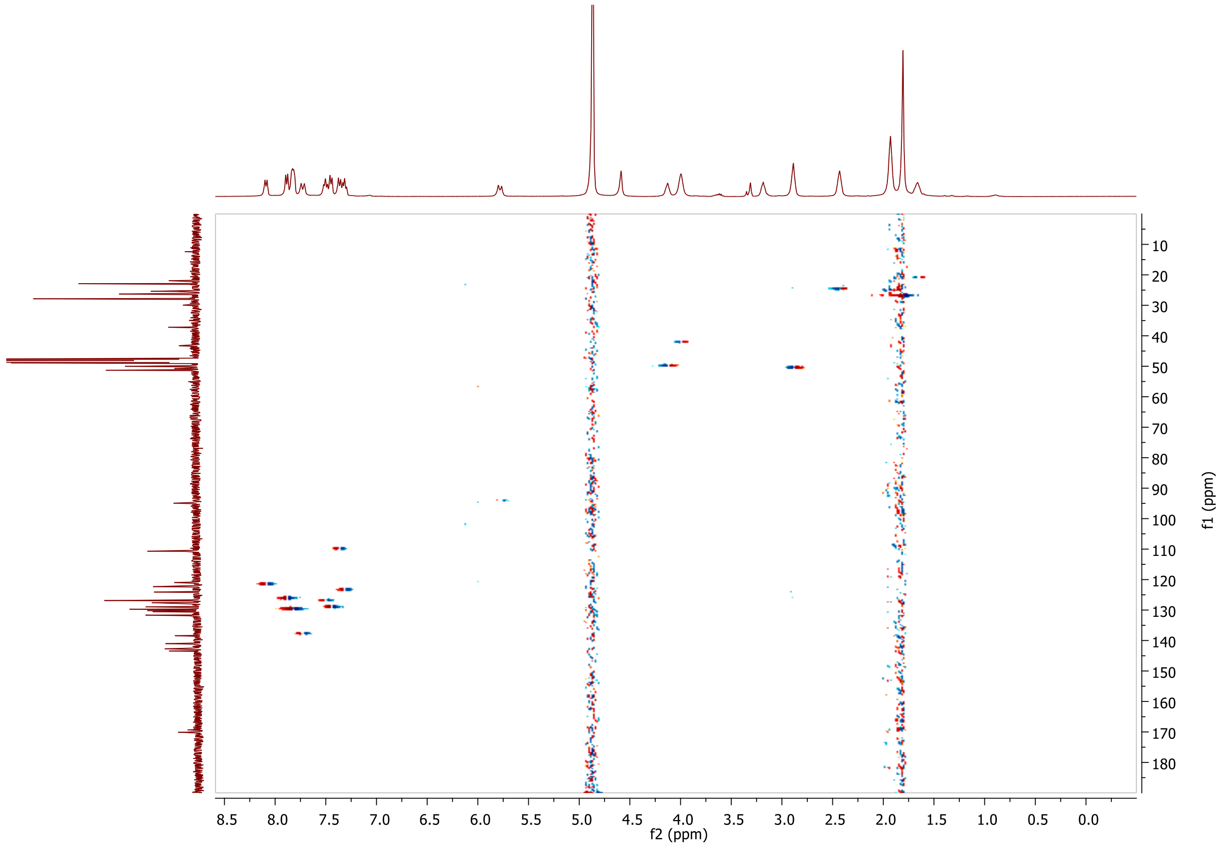

^1H-^{13}C HSQC spectrum (CD_3OD) of compound **2c** – 400 MHz

Carbonic Anhydrase activity

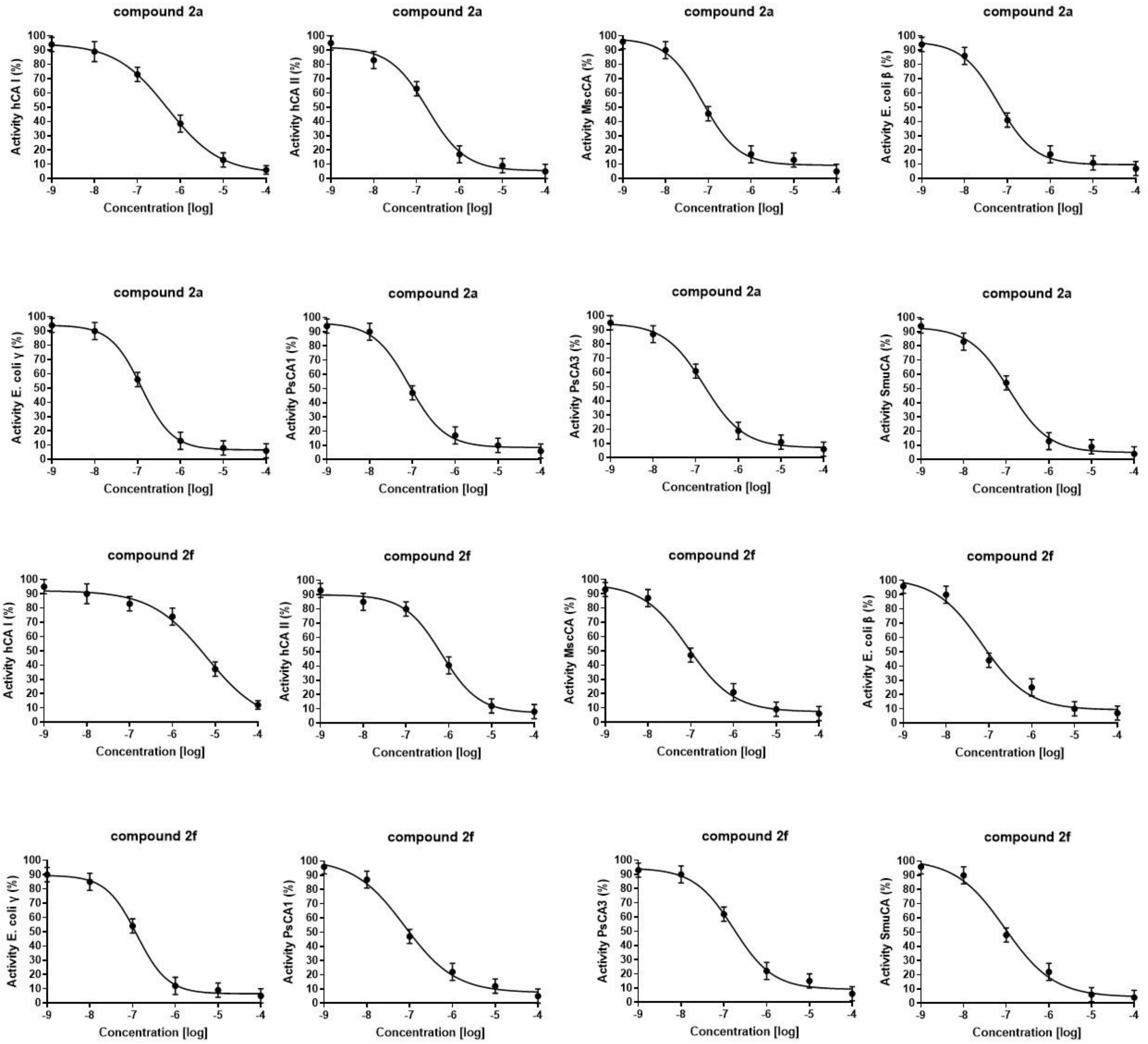

Supplement: Supplementary file 1 [file ijms-24-09610-s001.zip › ijms-2324664-supplementary.pdf]
